# Current advances and challenges in CAR T-Cell therapy for solid tumors: tumor-associated antigens and the tumor microenvironment

**DOI:** 10.1186/s40164-023-00373-7

**Published:** 2023-01-27

**Authors:** Ting Yan, Lingfeng Zhu, Jin Chen

**Affiliations:** 1 Institute of Clinical Medicine, The Second Affiliated Hospital of Hainan Medical University, Haikou, 570311 Hainan China; 2 Department of Urology, The Second Affiliated Hospital of Hainan Medical University, Haikou, 570311 Hainan China; 3 Department of Clinical Laboratory, The Second Affiliated Hospital of Hainan Medical University, Haikou, 570311 Hainan China

**Keywords:** CAR T cells, Solid tumor, TAAs, TSAs, TMAs

## Abstract

The past decade has witnessed ongoing progress in immune therapy to ameliorate human health. As an emerging technique, chimeric antigen receptor (CAR) T-cell therapy has the advantages of specific killing of cancer cells, a high remission rate of cancer-induced symptoms, rapid tumor eradication, and long-lasting tumor immunity, opening a new window for tumor treatment. However, challenges remain in CAR T-cell therapy for solid tumors due to target diversity, tumor heterogeneity, and the complex microenvironment. In this review, we have outlined the development of the CAR T-cell technique, summarized the current advances in tumor-associated antigens (TAAs), and highlighted the importance of tumor-specific antigens (TSAs) or neoantigens for solid tumors. We also addressed the challenge of the TAA binding domain in CARs to overcome off-tumor toxicity. Moreover, we illustrated the dominant tumor microenvironment (TME)-induced challenges and new strategies based on TME-associated antigens (TMAs) for solid tumor CAR T-cell therapy.

## Background

In the last decade, scientists and clinicians have come to recognize the great value of chimeric antigen receptor (CAR) T-cell therapy in the fight against hematological malignancies. Since 2017, numerous CAR T-cell therapies have been approved and/or reached clinical validation trials for hematological tumors by targeting CD19, CD20, CD22, and the B-cell maturation antigen (BCMA) [1–10]. As a novel class of therapies, CAR T-cell therapy has helped transform the treatment landscape for people with hematological malignancies [11, 12]. Currently, to further optimize CAR T-cell systems, scientists are focusing on CAR structure design, transfection, and cell culture techniques. Many of these techniques have reached clinical validation trials [13–16].

Because of its high remission rates, rapid tumor eradication, and long-lasting response in patients, CAR T-cell therapy has shown great therapeutic potential not only for hematological malignancies but also for solid tumors [17, 18]. However, the use of CAR T cells for solid tumor therapy remains a challenge due to the lack of specific antigens, off-target effects, and the complex tumor microenvironment [19]. In this review, we have outlined the development of the CAR T-cell technique, summarized the current tumor-associated antigens (TAAs) for CAR T-cell therapy in solid tumors, and highlighted the tumor-specific antigens (TSAs) or neoantigens and the TAA binding domain in CARs to overcome the off-tumor toxicity challenge. Furthermore, we illustrated the dominant challenges caused by the solid tumor microenvironment, including the immunosuppressive microenvironment, T-cell infiltration, antigen heterogeneity, and chronic stimulation. Combination with targeting tumor microenvironment antigens (TMAs) was proposed as a new strategy.

## Evolution of CAR design

Chimeric antigen receptors (CARs) are engineered proteins designed to enhance the tumor-targeting ability of immune cells. CARs have been used to generate various types of tumor-specific immune cells, such as T cells, NK cells, and macrophages. These immune cells, which are genetically modified through CAR transduction, are named CAR-T, CAR-NK, and CAR-M, respectively.

CAR is generally composed of an antigen-binding domain, a hinge region, a transmembrane region, one or more costimulatory domains, and an activation domain [11]. The extracellular antigen-binding (or recognition) domain consists of a single-chain variable fragment (scFv), which may also include peptides or other proteins that can recognize specific tumor antigens. The hinge region and the transmembrane region usually consist of either CD28 or IgG4. These proteins connect the intracellular and extracellular domains of the CAR. The costimulatory domains CD28 and 4-1BB (also known as CD137) are designed to increase T-cell proliferation, cytokine secretion, and the in vivo antitumor activity of CAR-T cells. The activation domain of CAR is made up of the CD3ζ domain, which transduces extracellular signals and activates T cells, leading to tumor lethality with cytolysis and cytokine release [20].

**Fig. 1:**
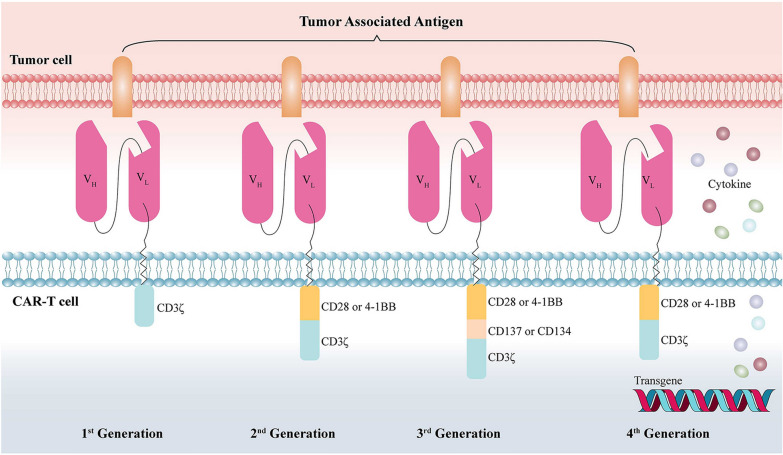
Four generations of CAR T cells. ​An overview of the four generations of CARs displayed on the surface of a T-cell while contacting their antigens on a tumor cell. scFvs that act as ligand-binding domains in CARs mediating tumor cell recognition are shown in red, with VH and VL domains connected to intracellular signaling domains via a hinge and a transmembrane domain

To date, the CAR T-cell technique has been developed for the fourth generation (Fig. 1). The first-generation CARs are formed from an scFv-based antigen-binding domain linked to a CD3ζ subunit of the T-cell receptor (TCR). Due to the lack of costimulatory signals, first-generation CARs are neither effective in amplification nor robust in antitumor activity, which limits their laboratory and clinical applications [21]. Compared with the first-generation CARs, the second-generation CARs include the whole structure of the first-generation CARs with the addition of costimulatory domains, such as CD28, 4-1BB, inducible T-cell costimulatory factor (ICOS), and DNA X-activating protein (DAP10) [21]. These modifications make CARs more favorable for inducing cytokine production and T-cell expansion, and some of these modifications also promote differentiation [22]. Third-generation CARs fuse CD137 or CD134 with CD28 simultaneously, which improves T-cell expansion and survival, promotes cytokine release, and enhances tumor killing [23]. Recently, the concept of fourth-generation CARs has been proposed. Fourth-generation CAR-T cells are further engineered to secrete universal cytokines, including IFN-γ, TNF-α, IL-2, and IL-8. The secretion of universal cytokines may enhance tumor-killing and immune cell recruitment effects [16, 21, 24–27].

## TAAs for CAR T-cell therapy in solid tumors

TAAs, which are highly expressed in tumor tissue and low or absent in normal tissue, are prerequisites for the application of CAR T cells to the treatment of solid tumors. They are selected based on pathology tests and immunohistochemistry results. After much effort by scientists, some specific antigens against solid tumors have been found to be applied to CAR T cells for solid tumor treatment.

### Mucin-1 (MUC1)

MUC1 is a tumor antigen closely related to tumorigenesis, invasion, and metastasis. A lentiviral vector containing the VH-based anti-MUC1 CAR gene is constructed by optimizing transient virus production followed by transfection into T cells to avoid immunogenic responses and signaling [28]. Transfected T cells were cocultured with MUC1-positive M47D or MCF-7 tumor cells or with MUC1-negative A431 tumor cells, and the cytotoxic activity of Th1 cytokines, including IL-2, TNF-α, and IFN-γ secreted by MUC1-recognizing CAR T cells, was significantly increased compared with that of untransduced T cells [28]. Heng Zhang et al. constructed enhanced CAR T cells, which not only targeted MUC1 but also activated JAK-STAT signaling induced by IL-12 and enhanced the proliferation of CAR T cells. The enhanced MUC1-CAR T cells possessed durable tumor-killing and proliferative capacity [29].

### B7H3 (CD276)

B7H3 is an immune checkpoint molecule expressed on the cell surface that plays an immune-inhibitory role in downregulating the biological activity of T cells and the cytotoxic activity of natural killer cells [30]. Its low expression in normal cells and abnormally high expression in a variety of malignancies suggest that B7H3 can be used as a novel target for CAR T-cell therapy [31]. Du H et al. found that B7H3 CAR T cells significantly inhibited pancreatic ductal cancer cells in vitro, while mice achieved 100% survival and showed no side effects [32]. B7H3 CAR T-cell injection (TAA06 injection), which was developed by PersonGen Co., Ltd., was approved for testing in clinical trials by the Center for Drug Evaluation (CDE) of the State Drug Administration for relapsed/refractory neuroblastoma on July 4, 2022.

### Human epidermal growth factor receptor 2 (HER2)

HER2 is a transmembrane glycoprotein that mediates cell proliferation and differentiation in both developing embryonic and adult tissues. HER2 can inhibit apoptosis, induce neovascularization, and enhance cell motility, thus promoting the rapid growth, proliferation, and metastasis of tumors [33, 34]. Sun et al. constructed humanized HER2-specific CAR T cells, which could react against HER2 + breast and ovarian cancer cells [35]. A phase I clinical trial (NCT01935843) demonstrated the safety and feasibility of HER2 CAR T cells in treating HER2-positive advanced biliary tract cancers and pancreatic cancers [36]. Moreover, HER2-targeted/4-1BB-costimulated CAR T cells could specifically recognize and kill synovial sarcoma cells by secreting IFN-γ and TNF-α [37]. Furthermore, HER2-specific CAR-T cells represent an effective immunotherapy for preventing CRC progression [38].

### Epidermal growth factor receptor (EGFR)

EGFR is expressed in normal cells but overexpressed in tumor cells [39]. Lin et al. showed that third-generation EGFR CAR T cells effectively inhibited triple-negative breast cancer with limited cytotoxicity to normal breast epithelial cells or estrogen receptor-positive breast cancer cells in vitro, and they performed in vivo experiments. In addition, EGFR CAR T cells activated the interferon γ, granzyme-perforin-PARP, and Fas-FADD-caspase signaling pathways in triple-negative breast cancer [40]. Liu et al. treated 16 patients with anti-EGFR CAR T cells, and the median overall survival of all 14 evaluable patients was 4.9 months, suggesting that anti-EGFR CAR T cells were safe and effective for the treatment of metastatic pancreatic cancer [41].

### Carcinoembryonic antigen (CEA)

CEA, a tumor-associated glycoprotein with a relative molecular mass of 180,000, is a tumor antigen produced continuously by progressive gastrointestinal malignancies. Investigators applied CAR T cells at five dose levels (1 × 10^5^ to 1 × 10^8^ CAR/kg cells) to 10 patients with colorectal cancer to demonstrate the safety and efficacy of treatment with CEA CAR T cells. Seven of these patients with progressive disease during previous treatments were stable after CAR T-cell therapy, and two were stable for over 30 weeks, showing tumor reduction by positron emission tomography (PET)/computed tomography (CT) and MRI analysis. The CAR T cells were observed to proliferate, especially after the second treatment [42]. Chi et al. designed CEA-specific CAR T cells in combination with rhIL-12 and evaluated their efficacy for treating different types of solid tumors. The in vivo results confirmed that the combination of CEA CAR T cells with rhIL-12 significantly enhanced their antitumor activity in terms of growth inhibition of the newly colonized colorectal cancer cell line HT-29, the pancreatic cancer cell line AsPC-1, and the gastric cancer cell line MGC803 compared with CEA CAR T-cell therapy alone. The combination of CEA CAR T cells and rhIL-12 overcame their separate defects to provide a new strategy for the treatment of solid cancer [43].

### Mesothelin (MSLN)

MSLN is a cell surface antigen involved in tumor invasion and is expressed at low levels in mesothelial tissues (pleural, pericardial, and peritoneal mesothelial cells) but is highly expressed in mesothelioma, pancreatic cancer, ovarian adenocarcinoma (OVCA), lung cancer, breast cancer, and other cancers, so it can be used as a target for CAR T cells in treating solid tumors [44–46]. Haas et al. constructed a second-generation CAR targeting MSLN in transiently expressing autologous T cells and tested them in a phase I study of toxicity. A patient with malignant pleural mesothelioma developed anaphylaxis during the second infusion of CAR T cells, presumably due to immune rejection of the murine-derived scFv in the CAR. This approach was safe overall but had limited therapeutic efficacy for other patients. Given the lack of targeted toxicity by RNA CAR T cells, the team conducted a phase I trial using lentiviral vector transduction. Overall, all patients with CAR T-meso cells had successful amplification but with a limited duration, and no targeted toxicity was observed, including pleurisy, peritonitis, or pericarditis [47]. MSLN CAR T-cell therapy is feasible, and researchers have found good antitumor activity in preclinical studies, but clinical data to date indicate that mesothelin CAR T cells are not effective in patients or do not proliferate consistently. Thus, new solutions should be sought.

### GD2

GD2, a disialoganglioside and acidic glycolipid found on the outer cell membrane, is a part of the immunological identity of mammalian cells and is generally nonimmunogenic. GD2 is expressed by a variety of embryonal cancers, including brain tumors, but it is barely expressed in normal cells [48], making it a target for CAR T therapy. Malvina et al. established a 3D culture glioblastoma model and reported that the intracerebral administration route significantly increased the survival rate in a dose-dependent manner without any side effects [49]. Researchers from Britain reported a phase 1 study (NCT02761915) in which 12 children with relapsed/refractory neuroblastoma were treated with escalating doses of second-generation GD2-directed CAR T cells. Their study showed that CAR T cells achieved rapid clearance of cancer. Although the beneficial effect lasted for only a short time, it provided important evidence that this particular CAR T-cell therapy could serve as a future treatment for solid cancers in children [50]. Recently, Majzner et al. carried out a clinical trial (NCT04196413) of autologous GD2 CAR T cells for the treatment of H3K27 M-mutated diffuse midline gliomas (DMGs) in children and young adults, identifying the maximally tolerated dose or recommended phase II dose. The tumor progressed in one of the four enrolled patients, and the patient died due to disease progression after three months. The other three patients derived radiographic and clinical benefits after i.v. administration of GD2 CAR T cells, although they developed CRS and tumor inflammation-associated neurotoxic reactions. This study provided valuable experience for further optimizing GD2 CAR T-cell therapy for spinal DMG [51].

### Natural killer group 2D (NKG2D)

NKG2D-based CAR T cells have received much attention in the past few years because of their ability to recognize a panel of eight stress-associated ligands in the MIC and ULBP families, which are hardly expressed on normal tissues but are upregulated in malignant transformation, viral infection, and DNA damage. Tay et al. manufactured NKG2D CAR T cells with piggyBac transposon vectors and K562 artificial antigen-presenting cells instead of viral vectors. The results showed that small volumes of peripheral blood samples could produce many NKG2D CAR T cells, and they could be expanded to meet a clinical timeline designed for solid cancer therapy [52]. YanZhang designed an NKG2D CAR T-cell-fused NKG2D extracellular domain and 4-1BB and CD3z, which showed a strong antitumor effect against NKG2DL-positive cervical cancer cell lines in vitro as well as xenograft mice in vivo without toxicity [53]. Yu-Yang Ng constructed NKG2D CAR T cells with a DAP12 signaling domain that stimulated lower cytokine production to mitigate the risk of CRS compared with the CD3ζ activation domain but displayed similar antitumor activity, which completely eradicated established solid tumor xenografts in an NSG mouse model [54].

### Epithelial cell adhesion molecule (EpCAM)

​EpCAM is a type I transmembrane glycoprotein that is frequently overexpressed in carcinomas, including colorectal, gastric, pancreatic, and endometrial cancers, in a heterogeneous manner [55, 56]. It was first found in colon cancer tissue and is a target of the Wnt/β-catenin pathway, the activation of which frequently contributes to poor infiltration of T cells across most human cancers [57]. Juan Fu found that the EpCAM target is highly upregulated in actual ovarian cancer samples and sought to construct 3rd -generation EpCAM CAR T cells and then verified their antitumor activities in vitro and in vivo. Their results showed that EpCAM CAR T cells could become a clinical therapeutic strategy against ovarian cancer [56]. There are an increasing number of clinical trials registered utilizing EpCAM CAR T cells (NCT04151186, NCT02915445, NCT03013712).

Numerous types of TAAs for CAR T targets are tested in clinical trials, not only those referenced above that have been tested in at least three trials thus far (MUC1, B7H3, HER2, EGFR, CEA, MSLN, GD2, NKG2D, and EpCAM) but also Nectin4, FAP, TM4SF1, LOH, CD22/TILs, claudin 18.2, ROR2, CLDN6, Lewis Y (LeY), glypican 3, PSCA, ROR1, CD7, IM96, IM92, IL13α2, GPC3, Mov19-BBz, CT041, PSMA, GUCY2C, and others. To date, 125 solid tumor CAR T clinical studies have been registered at ClinicalTrials.gov worldwide as of September 07, 2022 (Table 1). Moreover, hundreds of potential targets for CAR T-cell therapy are being applied to solid tumors in preclinical trials.

**Table 1:** The clinical trials of CAR T cell target to solid tumors^a^

Antigen	Items	Conditions or disease	Intervention/treatments	Phase	NCT number
MSLN	17	Colorectal Cancer, Ovarian Cancer, Advanced Solid Tumor, Mesothelioma, Metastatic Liver Cancer, Cervical Cancer, Pancreatic Cancer, Ovarian Cancer, Mesothelioma, Lung Cancer, Gut Microbiota	αPD1-MSLN-CAR T cells; CTLA-4/PD-1 antibodies expressing mesoCAR-T; Mesothelin-directed CAR T cells; RD133; Fludarabine; Aldesleukin	I, II	NCT04503980, NCT05089266, NCT03182803, NCT04489862, NCT03747965, NCT03545815, NCT03030001, NCT03615313, NCT05166070, NCT01583686, NCT02930993, NCT04981691, NCT03323944, NCT05373147, NCT05141253, NCT05344976, NCT03941626
B7H3 (CD276)	9	Pediatric Solid Tumor, Germ Cell Tumor, Retinoblastoma, Hepatoblastoma, Wilms Tumor, Rhabdoid Tumor, Carcinoma, Osteosarcoma, Ewing Sarcoma, Rhabdomyosarcoma, Neuroblastoma, Adrenocortical Cancer, Desmoplastic Small Round Cell Tumor, Gastric Cancer, Lung Cancer, Ovarian Cancer	Second generation 4-1BBζ B7H3-EGFRt-DHFR (selected) and a second generation 4-1BBζ CD19-Her2tG; Targeting CD276 autologous chimeric antigen receptor T cells; Fludarabine, Cyclophosphamide, MESNA, B7-H3 CAR T cells; EGFR/ B7H3-positive Advanced Lung Cancer, EGFR/ B7H3-positive Advanced Triple-negative Breast Cancer; Fludarabine, Cyclophosphamide	I, II	NCT04483778, NCT04691713, NCT04897321, NCT04864821, NCT05211557, NCT05323201, NCT04670068, NCT05515185, NCT05190185
EGFR	8	PD-1 Antibody, CAR-T Cells, Advanced Solid Tumor, Pediatric Solid Tumor, Germ Cell Tumor, Retinoblastoma, Hepatoblastoma, Wilms Tumor, Rhabdoid Tumor, Carcinoma, Osteosarcoma, Ewing Sarcoma, Rhabdomyosarcoma, Advanced Solid Tumor, PD-1 Antibody, CAR-T Cells, Advanced Malignancies, Solid Tumor, Adult, EGFR Overexpression, Non Small Cell Lung Cancer, Biological: CXCR5 modified EGFR Chimeric Antigen Receptor Autologous T cells	HerinCAR-PD1 cells; second generation 4-1BB#, EGFR806-EGFRt, second generation 4-1BB#, EGFR806-EGFRt and a second generation 4 1BB# CD19-Her2Tg; anti-CTLA-4/PD-1 expressing EGFR-CAR-T; HerinCAR-PD1 cells; EGFR/ B7H3-positive Advanced Lung Cancer, EGFR/ B7H3-positive Advanced Triple-negative Breast Cancer	I, II	NCT02862028, NCT03618381, NCT03182816, NCT02873390, NCT04976218, NCT04153799, NCT05060796, NCT01218867
GD2	7	Neuroblastoma, Neuroblastoma Recurrent, GD2-positive Solid Tumors, Osteosarcoma, Ewing Sarcoma, Sarcoma, Melanoma, GD2 Positive Glioma, CAR-T Cell Immunotherapy	GD2-CART01; Anti-GD2-CAR engineered T cells, AP1903, Cyclophosphamide; CAR-T cell Immunotherapy; iC9-GD2 T Cells – frozen, iC9-GD2 T Cells – fresh, Cytoxan, Fludara, Keytruda, iC9-GD2 T cells; 4SCAR-GD2; iC9.GD2.CAR.IL-15 T cells, Cyclophosphamide, Fludarabine;	I, II	NCT03373097, NCT02107963, NCT03252171, NCT01822652, NCT02992210; NCT03721068, NCT03635632
HER2	6	Gastric Cancer, Breast Cancer, Ovarian Cancer, Sarcoma, Bladder Cancer, Head and Neck Squamous Cell Carcinoma, Cancer of the Salivary Gland, Lung Cancer, Esophageal Cancer, Colorectal Cancer, Pancreatic Adenocarcinoma, Glioma, Bile Duct Cancer, Biliary Tract Cancer, Peritoneal Carcinoma Metastatic, Pleural Effusion, Malignant, Sarcoma	CAR T cell therapy; CCT303-406; CAdVEC; Anti-HER2 CAR-T; CT-0508; Dual-targeting HER2 and PD-L1 CAR-T cells; cells; Pembrolizumab Injectable Product, Nivolumab Injectable Product, Lymphodepletion Chemotherapy	I, II	NCT04650451, NCT04511871, NCT03740256, NCT02713984, NCT04660929, NCT04684459, NCT04995003
MUC1	7	Advanced Solid Tumor, Breast Cancer, Ovarian Cancer, Non Small Cell Lung Cancer, Colorectal Cancer, Pancreatic Cancer, Renal Cell Carcinoma, Nasopharyngeal Cancer, Head and Neck Squamous Cell Carcinoma, Gastric Cancer, Fallopian Tube Cancer, Triple Negative Breast Cancer, Multiple Myeloma, Pancreatic Ductal Adenocarcinoma, Hepatocellular Carcinoma, Non-small Cell Lung Cancer, Malignant Glioma of Brain, Advanced Esophageal Cancer	Anti-CTLA-4/PD-1 expressing MUC1-CAR-T; P-MUC1C-ALLO1 CAR-T cells, Rimiducid, CART-TnMUC1, Cyclophosphamide, Fludarabine, anti-MUC1 CAR-pNK cells	I, II	NCT03179007, NCT02587689, NCT05239143, NCT04025216, NCT02839954, NCT03706326, NCT02617134
NKG2D	6	Hepatocellular Carcinoma, Glioblastoma, Medulloblastoma, Colon Cancer, Colorectal Cancer, Triple Negative Breast Cancer, Sarcoma, Nasopharyngeal Carcinoma Prostate Cancer, Gastric Cancer, Glioma, Malignancy, Refractory Cancer, Relapsed Cancer, CRC	NKG2D-based CAR T-cells; Adoptive Cell Transfer of NKG2DL-targetting Chimeric Antigen Receptor-grafted Gamma Delta T cell; KD-025 CAR-T cells	I	NCT05131763, NCT04270461, NCT04107142, NCT05302037, NCT05382377, NCT04550663
CEA	6	Lung Cancer, Colorectal Cancer, Liver Cancer, Pancreatic Cancer, Gastric Cancer, Breast Cancer, Esophageal Cancer, Stomach Cancer, Metastatic Tumor, Recurrent Cancer	CEA CAR-T cells, Anti-CEA CAR-T cells, gemcitabine/nab paclitaxel, NLIR + FU/FA, Capecitabine	I, II, III	NCT04348643, NCT03818165, NCT04037241, NCT05396300, NCT05415475, NCT05240950
EpCAM	2	Advanced Solid Tumor, Neoplasms, Malignant Neoplasm of Nasopharynx TNM Staging Distant Metastasis (M), Breast Cancer Recurrent, Colon Cancer, Esophageal Carcinoma, Pancreatic Cancer, Prostate Cancer, Gastric Cancer, Hepatic Carcinoma	TM4SF1- and EpCAM-positive chimeric antigen receptor T-cell therapy; CAR-T cells recognizing EpCAM	I, II	NCT02915445, NCT03013712
Nectin4/FAP	1	Nectin4-positive Advanced Malignant Solid Tumor	Biological: CAR-T therapy for nectin4-positive malignant solid tumor	I	NCT03932565
LOH	1	Solid Tumor, Adult Colorectal Cancer, Non Small Cell Lung Cancer, Pancreatic Cancer, CRC, NSCLC, Pancreas Cancer	Other: Leukapheresis, Diagnostic Test: Next Generation Sequencing (NGS), Diagnostic Test: Long Range NGS HLA typing	NA	NCT04981119
CD22	1	Solid Tumor, Adult, Cervical Cancer, Sarcoma, NSCLC	Biological: Autologous aPD-L1 armored anti-CD22 CAR T cells	I	NCT04556669
Claudin18.2	8	Advanced Solid Tumor, Gastric Cancer, Pancreatic Ductal Adenocarcinoma	Drug: CAR-CLDN18.2 T-Cells, Drug: PD-1 Monoclonal Antibody, Drug: Chemotherapy, Drug: Cell injection, Drug: IBI345	I	NCT03874897, NCT05284968, NCT03890198, NCT04467853, NCT05472857, NCT05393986, NCT05199519, NCT04966143
CLDN6	1	Solid Tumor	Biological: CLDN6 CAR-T, Biological: CLDN6 RNA-LPX	I,II	NCT04503278
LeY	1	Advanced Cancer	Biological: LeY CAR T cells	I	NCT03851146
Glypican 3	5	Liver Cancer, Rhabdomyosarcoma, Malignant Rhabdoid Tumor, Liposarcoma, Wilms Tumor, Yolk Sac Tumor	Genetic: AGAR T cells, Drug: Cytoxan, Genetic: CARE T cells, Drug: Cytoxan, Genetic: GAP T cells, Drug: Cytoxan, Drug: Fludara	I	NCT04377932, NCT05103631, NCT04715191, NCT02932956, NCT04093648
PSCA	1	Metastatic Castration-resistant Prostate Cancer, Metastatic Prostate Cancer, Metastatic Pancreatic Ductal Adenocarcinoma, Metastatic Pancreatic Cancer, Metastatic Pancreatic Adenocarcinoma	Biological: BPX-601, Drug: Rimiducid	I, II	NCT02744287
ROR1	1	Triple Negative Breast Cancer, TNBC-Triple-Negative Breast Cancer, Non-small Cell Lung Cancer, Non Small Cell Lung Cancer Metastatic, Non-Small Cell Carcinoma of Lung, TNM Stage 4, Advanced Breast Cancer, Advanced Lung Carcinoma, NSCLC, Recurrent, and 5 more	Biological: LYL797	I	NCT05274451
Treatment for CRS	1	Solid Tumor, Hematological Malignancy	Drug: Metoprolol, Drug: metoprolol, infliximab, etanercept, tocilizumab and/or other agents	I, II	NCT04082910
PSMA	4	Bladder Cancer, Urothelial Carcinoma Bladder, Prostatic Neoplasms, Castration-Resistant, Neoplasms by Histologic Type, Neoplasms, Prostate Cancer, Metastatic Castration-resistant Prostate Cancer, Neoplasms, Genital Neoplasms, Male, Urogenital Neoplasms, Neoplasms by Site and 7 more	Genetic: 4SCAR-PSMA, Genetic: 4SCAR-Fra, P-PSMA-101 CAR-T cells, Drug: Rimiducid, Drug: Cyclophosphamide (Non-IMP), Drug: Fludarabine (Non-IMP), Drug: UniCAR02-T-pPSMA, Drug: UniCAR02-T (IMP)	I, II	NCT03185468, NCT04249947, NCT04429451, NCT04633148
Targeting Cervical cancer	1	Cervical Cancer	Biological: Cervical cancer-specific CAR-T cells	I, II	NCT03356795
Targeting Pancreatic Cancer	2	Pancreatic Cancer, CAR Platinum-Resistant Fallopian Tube Carcinoma, Platinum-Resistant Ovarian Carcinoma, Platinum-Resistant Primary Peritoneal Carcinoma, Recurrent Fallopian Tube Carcinoma, Recurrent Ovarian	Drug: Chimeric antigen receptor T cell	I	NCT03267173, NCT03323944
PRGN-3005	1	Tube Carcinoma, Recurrent Ovarian Carcinoma, Recurrent Primary Peritoneal Carcinoma, Refractory Fallopian Tube Carcinoma, Refractory Ovarian Carcinoma, Refractory Primary Peritoneal Carcinoma, Stage III Fallopian Tube Cancer AJCC v8 and 24 more	Biological: PRGN-3005 UltraCAR-T cells	I	NCT03907527
IM96	1	Advanced Solid Tumors, Digestive System Neoplasms, Pancreatic Cancer Resectable, Colorectal (Colon or Rectal) Cancer	Drug: IM96 CAR-T cells	I	NCT05287165
IM192	1	Advanced Solid Tumors, Gastric Cancer, Esophagogastric Junction Cancer, Pancreatic Cancer	Drug: IM92 CAR-T cells	I	NCT05275062
IL13Ralpha2	1	Clinical Stage IV Cutaneous Melanoma AJCC v8, IL13RA2 Positive, Metastatic Melanoma, Cutaneous Melanoma, Stage III, Cutaneous Melanoma, Stage IV	Drug: Cyclophosphamide, Drug: Fludarabine Phosphate, Biological: IL13Ralpha2-specific Hinge-optimized 4-1BB-co-stimulatory CAR/Truncated CD19-expressing Autologous TN/MEM Cells, Drug: Recombinant Interleukin-2	I	NCT04119024
Targeting Sarcoma	2	Sarcoma, Osteoid Sarcoma, Ewing Sarcoma	Biological: Sarcoma-specific CAR-T	I, II	NCT03356782, NCT04433221
GPC3	1	Carcinoma, Hepatocellular	Drug: TAI-GPC3-CART cells	I, II	NCT02715362
Targeting Malignant Glioma	1	Glioma, Malignant Glioma of Brain, Recurrence Tumor	Biological: chimeric antigen receptor T cells	I	NCT03423992
MOv19-BBz	1	Ovarian Cancer, Fallopian Tube Cancer, Primary Peritoneal Carcinoma	Drug: MOv19-BBz CAR T cells, Device: Alpha Folate Receptor expression test	I	NCT03585764
CT041	1	Malignant Neoplasms of Digestive Organs	Biological: gucy2c cart cells	I	NCT04652219, NCT04780529
Gucy2c	2	Malignant Neoplasms of Digestive Organs	Biological: gucy2c cart cells	I	NCT04652219, NCT04780529
TM4SF1/EpCAM	1	Advanced Solid Tumor, Neoplasms	Biological: TM4SF1- and EpCAM-positive CAR T-cell therapy	NA	NCT04151186
CD70	3	Metastatic Tumor, Advanced Solid Tumor, Renal Cell Carcinoma, Ovarian Cancer, Cervix Cancer	Biological: CD70 CAR-T cells	I	NCT05468190, NCT05518253, NCT05420545
GD2/PSMA	1	Solid Tumor	Biological: bi-4SCAR GD2/PSMA T cells	I, II	NCT05437315
EGFR/B7H3	1	EGFR/ B7H3-positive Advanced Lung Cancer, EGFR/ B7H3-positive Advanced Triple-negative Breast Cancer	Biological: EGFR/B7H3 CAR-T	I	NCT05341492
ROR2	1	Solid Tumor, Soft Tissue Sarcoma, Gastric Cancer, Pancreatic Cancer, Bladder Cancer	Biological: CCT301-59	I	NCT03960060
OX40	1	Lung Cancer, Hepatocellular Carcinoma, Solid Tumor	Combination Product: CpG-ODN	I	NCT04952272
HER2/PD-L1	1	Peritoneal Carcinoma Metastatic, Pleural Effusion, Malignant	Biological: Dual-targeting HER2 and PD-L1 CAR-T cells	I	NCT04684459
VEGFR1/PD-L1	1	Malignant Peritoneal Effusion, Malignant Ascites, Serous Cavity Metastatises	Biological: Dual-targeting VEGFR1 and PD-L1 CAR-T cells	I	NCT05477927
Effects on PC by gut microbiota regulation	1	Pancreatic Cancer, Gut Microbiota, CAR-T	NA	NA	NCT04203459
PD-L1	1	Glioblastoma Multiforme	Biological: Anti-PD-L1 CSR T cells, Drug: Cyclophosphamide, Drug: Fludarabine	I	NCT02937844
CD44v6	1	Cancers Which Are CD44v6 Positive	Biological: CD44v6-specific CAR gene-engineered T cells	I, II	NCT04427449
EBV	1	Nasopharyngeal Carcinoma	Biological: EBV-TCR-T (YT-E001) cells	II	NCT03648697
NY-ESO-1	1	Lung Cancer, Nonsmall Cell Recurrent	Drug: Cyclophosphamide and Fludarabine, Biological: Anti-NY-ESO-1 TCR	I	NCT03029273
GD2/CD70	1	Cancer Disease	Biological: bi-4SCAR GD2/CD70 T cells	I, II	NCT05438368
MUC16ecto	1	Solid Tumors	Procedure: Production of Genetically-modified T cells, Drug: Cyclophosphamide, Device: IP Catheter Insertion, Genetic: Infusion of 4H11-28z/fIL-12/EGFRt+ Genetically-modified T cells, Drug: Fludarabine	I	NCT02498912

^a^The clinical trials data was collected from clinicaltrials.gov. There were 125 studies clinical trials about CAR-T treatment for solid tumor in the clinicaltrials.gov as of September 07, 2022

## Tumor neoantigens for CAR T-cell therapy in solid tumors

Tumor neoantigens, which are tumor-specific antigens derived from somatic mutations that are expressed only on tumor cells and are more immunogenic and have less off-tumor toxicity than TAAs [58], may represent a new approach for CAR T-cell therapy.

Tumor neoantigens have been employed in the development of tumor vaccines for solid tumor treatment due to their specific immunogenicity. To date, hundreds of peptide vaccines for solid tumors based on tumor neoantigens have reached clinical validation trials. A typical example is the personalized neoantigen peptide vaccine for the treatment of EGFR-mutated non-small cell lung cancer, which has reached phase I clinical trials. Additionally, there are immune cells that are expanded in vitro targeting tumor neoantigens for solid tumor treatment, such as neoantigen-induced autologous DC-CIK cells (NCT05020119) and tumor-infiltrating lymphocytes (TILs) for terminal epithelial tumors (NCT05141474).

At present, CAR T cells targeting tumor neoantigens for solid tumor treatment have been developed. A case in point is that anti-EGFRvIII-CAR T cells can efficiently kill EGFRvIII cancer cells, which are high-grade glioblastoma cells with specific TSAs [59]. You Li’s team optimized an EGFRvIII-specific CAR construct with the TGFRII ectodomain as a TGFβ-resistant CAR-T for glioblastoma therapy, and the results showed that the architecture enhanced the antitumor efficacy of EGFRvIII-specific CAR-T cells and prolonged the survival of glioblastoma-bearing mice [60]. Bryan et al. developed bispecific CAR structures specifically targeting the glioblastoma tumor antigen EGFRvIII and bispecific T-cell engager (BiTE) against EGFR. BiTE cells secreted EGFR-specific BiTEs and recruited untransformed bystander T cells against wild-type EGFR. These CAR T BiTE cells eliminated heterogeneous tumors in a glioblastoma mouse model [61]. Several CAR T cells targeting EGFRvIII for recurrent glioblastoma treatment have reached clinical trials (NCT02844062 and NCT01454596).

## Challenges of using TAAs for CAR-T-cell therapy in solid tumors

### On target, off-tumor toxicity

Although many TAAs have been selected for CAR-T-cell therapy, and some of them have shown gratifying outcomes, off-tumor toxicity remains a major challenge because TAAs may also be expressed in normal tissues. For example, TAAs such as HER2 and EGFR are overexpressed in cancer cells, but they are also expressed at low density in normal epithelial tissues. Therefore, some experiments have shown that anti-HER2 CAR T cells are toxic to normal organs. This off-tumor target toxic effect is also observed in other CARs targeting the overexpression of TAAs, and severe off-tumor target effects may result in patient death [62]. Therefore, the design of CAR-T cells needs to be able to sense the density of antigens to distinguish between cancer cells and normal cells.

### Lack of public tumor neoantigens

To date, although TSAs and tumor neoantigens are the ideal antigens for developing efficient CAR T cells, only a few TSAs in their true sense have been reported, such as BRAF, KRAS, TP53, HRAS/KRAS/NRAS, and BRAF [63]. These reported public TSAs can be used to generate TCR, CAR T cells, and dual-specific antibodies. Nevertheless, the downstream signals are different due to the differences in the formation of immune synapses, affinity, and structural dynamics of TCR, CAR T cells, and bispecific antibodies. ​In addition, these antigens are low in content, typically less than 100 copies per cell, whereas CAR T typically requires hundreds to thousands of copies for effective identification [63]. Therefore, it is urgent to establish prediction and identification methods for TSAs. Neoantigens can be discovered by genomic techniques such as next-generation sequencing and high-throughput single-cell sequencing, MHC-loaded peptide techniques such as mass spectrometry, and bioinformatics tools based on high-throughput sequencing data, mass spectrometry data, and biological databases [64–66]. The abovementioned techniques are essential for the discovery of TSAs, which may boost the development of CAR T-cell therapy for solid tumor treatment.

### Tumor-antigen heterogeneity

The heterogeneity of TSAs is another key factor that leads to immunologic escape and tumor recurrence [67]. Approximately 30% of glioblastomas express mutant EGFRv III. A clinical trial demonstrated that the treatment of gliomas using anti-EGFRvIII CAR T-cell therapy leads to the growth of EGFRvIII-negative tumor cells [67]. Tumor-antigen heterogeneity is a challenge to overcome that results in unsatisfactory CAR T-cell therapy of solid tumors. To promote the effectiveness of CAR T-cell therapy, new strategies for tumor recognition are needed to cope with the challenges of tumor specificity and heterogeneity.

## Strategies for overcoming the challenges of TAAs

### Dual CAR and tandem CAR

The antigen of CARs requires specificity to recognize tumor cells, avoiding off-target effects. Additionally, to eliminate the possibility of tumor recurrence resulting from immunological escape, the antigen also needs to be universal to overcome the heterogeneity of tumor cells. Combining two or more antigens as targets and constructing multitarget CARs are potential approaches to solve the contradiction between specificity and heterogeneity.

Bispecific CARs have become an effective way to avoid tumor relapse due to antigen escape. They contain two different CARs coexpressed on one T cell, called dual CARs. Dual-targeting CAR T cells can be precisely positioned at the tumor site, have high cytotoxicity to tumor cells, alleviate off-target effects, and achieve precise treatment [68]. For example, the tumor-specific antigen EGFRvIII is activated by the SynNotch receptor to induce local CAR expression, which is then coupled with EphA2 and IL13Rα2 to induce tumor cell recognition and killing. This approach may avoid the off-target killing of the TAAs EphA2 and IL13Rα2 in distant normal tissues [69]. Peng Li’s team developed a DAP10 chimeric receptor with native NKG2D on T cells to target NKG2D ligand-expressing cancer cells. They tandemly incorporated it with anti-glypican 3 (GPC3) scFv to construct a dual-antigen-targeting system. This novel dual-targeting system enabled high efficacy in killing heterogeneous cancer tumors [70]. Bob S. Carter et al. designed EGFRvIII and BiTE bicistronic construct CAR T cells against EGFR to solve heterogeneous target antigen expression. Tumors lacking the antigen targeted by CAR T cells directed against single antigens grew outward [61]. Therefore, it is essential to recognize multiple antigens in tumor cells with a single CAR T-cell or to coexpress various CAR proteins and to achieve programmable CAR expression regulation with individual T cells.

Two CAR T-cell lines, each targeting a different antigen, were combined and named the CAR pool. T cells expressing a single-chain bispecific CAR are known as tandem CAR. HER2/MUC1 bispecific CAR for breast cancer cell therapy in vitro [71], an EGFR/EpCAM/HER2 triple-specific CAR against Raji lymphoma cells engineered to express TAAs [72], and a HER2/IL-13Ra2 bispecific CAR for the treatment of a glioma xenograft in vivo have shown promising results [73]. Dual GD2 and B7H3 CAR T cells provided optimal costimulation and T-cell metabolic fitness by independently activating the CD28 and 4-1BB pathways and fine-tuning CD3ζ chain-mediated signaling in a neuroblastoma model.

There are also studies of CEA plus MSLN dual-target CAR T cells expressing CEA-CD3ζ and the MSLN-4/1BB signaling domain. These dual-targeted CAR T cells became dCAR T cells, and dCAR T cells were significantly cytotoxic to two antigen-positive tumor cells but not to single antigen-positive tumor cells both in vivo and in vitro. The dual CAR T cells showed robust and sustained antitumor activity in vivo under stress conditions and prevented tumor escape due to heterogeneous antigen expression by tumor cells [74]. Sandra et al. constructed a dual-specific antibody, BiTE, which could target both EGFR of tumor cells and CD3 of T cells, and infused this BiTE into T cells, resulting in no attacks on normal tissues while locally killing tumor cells, indicating that this BiTE CAR T could well attenuate off-target effects [75].

### Improved recognition ability of the TAA binding domain in CARs

**Fig. 2:**
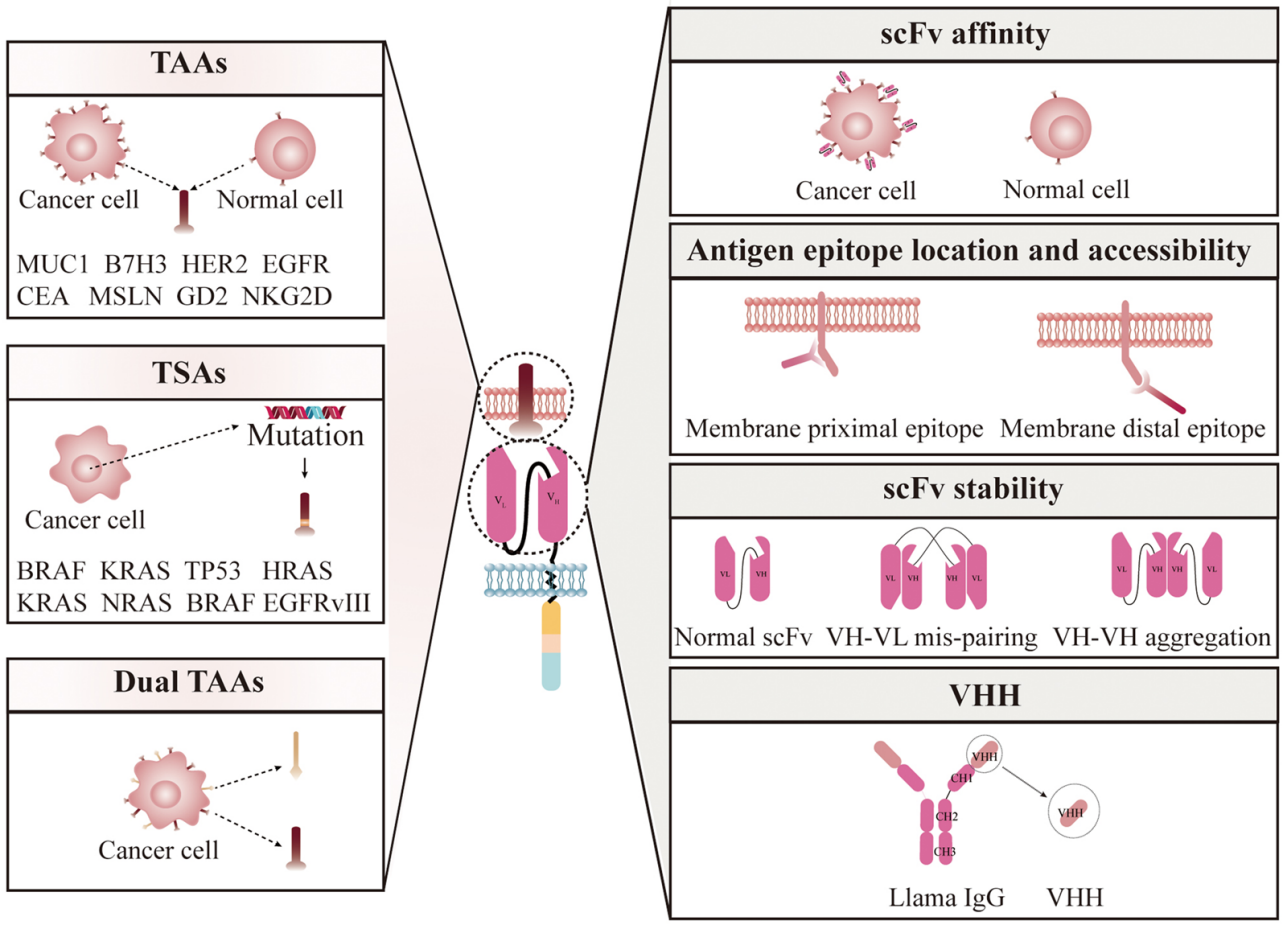
TAAs and TAA binding domain design strategy for developing multispecific CARs for solid tumors. TAAs and TSAs were selected as CAR T-cell targets. To overcome the challenges of tumor antigen off-target effects and heterogeneity, dual CARs or tandem CARs have been constructed to target dual TAAs. scFv affinity, steric hindrance, and stability were considered for the construction of CARs, and VHH nanobodies were applied as TAA binding domains to develop multispecific CARs for solid tumors

Another key factor for the antitumor effect of CAR T cells is the recognition ability of the TAA binding domain in CARs. The interaction between the CAR and its binding target determines the tumor-killing effect and proliferation capacity of the constructed CAR-T cells and, to some extent, the persistence of the response [76]. ​ The TAA binding domain can be adjusted to enhance the scFv affinity, scFv steric hindrance, and stability of the TAAs or nanoantibodies can be used to enhance the ability of the TAA binding domain to better recognize and bind the TAAs (Fig. 2).

​Because of their small size, high affinity and antigen-specific recognition, scFvs are widely applicable to CAR-T therapy and bispecific antibody development. Modulating the affinity of scFv may improve the selective identification of target cells with higher ligand densities, thereby reducing off-target effects. Because TAAs are expressed not only in tumors but also to some extent in normal cells, modulating the affinity of scFvs can help to increase CAR specificity and reduce off-target effects [77, 78].

​Alternatively, adjusting the steric hindrance of scFvs based on the structure and function of target epitopes on TAAs may improve the antitumor effect. Researchers have used the piggyBac transposon system to construct two types of CAR, CAR-T cells targeting region I (meso1, residues 296–390) or region III (meso3, residues 487–598) of MSLN, and meso3 CAR-T cells were found to produce more CD107α, IL-2, TNF-α, and IFN-γ relative to meso1 CAR-T cells upon activation and showed an enhanced killing effect [79]. Therefore, meso3 has a better-targeted epitope than meso1, which may be related to the specific characteristics of the proximal membrane region. When the proximal membrane region bridges the extracellular and transmembrane domains of the MSLN, it is likely to have a rigid structure or be responsible for a specific functional region, and when the scFv on CAR-T binds to this region, it triggers more effective antitumor activity.

Moreover, precise control of the expression of scFvs on CAR may improve the intrinsic stability of the binding domains of TAAs. If the amount of scFv expression is too small, the amount of binding to the TAA antigen epitope would be too small to effectively capture tumor cells and exert CAR-T-cell antitumor effects. In turn, too high expression of scFvs can lead to scFv aggregation and ultimately to early depletion of T cells [80].

### Nanobodies applied as the TAA binding domain of CAR-T cells

​Nanobodies, also known as VHH antibodies, are derived from the variable domain of a heavy chain antibody and can bind to antigen recognition sites. The nanobodies consist of a long CD3 sequence that accepts an adjusted flexible and extended conformation, capable of reaching certain epitopes that are inaccessible to conventional antibodies. In contrast to scFvs, VHH antibodies avoid potential disruption of interactions between variable (VH, VL) and constant (CH1, CL) regions and avoid exposure to hydrophobic regions, both of which can affect antibody solubility and stability [81]. Alternatively, nanotubes that act as TAA-binding domains for CARs do not produce surface aggregates and do not have target antigen-independent effects or cell activation limitations. Nanobodies with optimal stability and high affinity applied as the antigen binding domain of CAR-T cells may improve the specific binding of TAAs and CARs.

​As the extracellular binding domain of scFvs, it is difficult to balance optimal affinity, obtaining optimal steric hindrance, and optimal epitope and stability for targeting TAAs. The ability to generate functional multispecific CARs using nanobodies, D-domains, and other novel molecules with beneficial antitumor effects offers the opportunity to develop multispecific CARs that effectively address tumor recurrence due to antigen loss and provide a therapeutic approach to the intrinsic phenotypic diversity of tumors.

## Challenges originating from the tumor microenvironment

The tumor microenvironment is a complex and evolving entity that contains immune cells, stromal cells, blood vessels, cytokines, and the extracellular matrix, and its features continuously promote tumor progression [82]. ​Early in tumor initiation, the tumor microenvironment promotes early cancer cell growth, supports tumor survival, invasion, and spread, and promotes angiogenesis to overcome hypoxic and acidic environments [83]. The immunosuppressive tumor microenvironment will cause CAR T cells to infiltrate into solid tumors inefficiently, resulting in inadequate activation and exhaustion of CAR T cells.

### Tumor immunosuppressive microenvironment

**Fig. 3:**
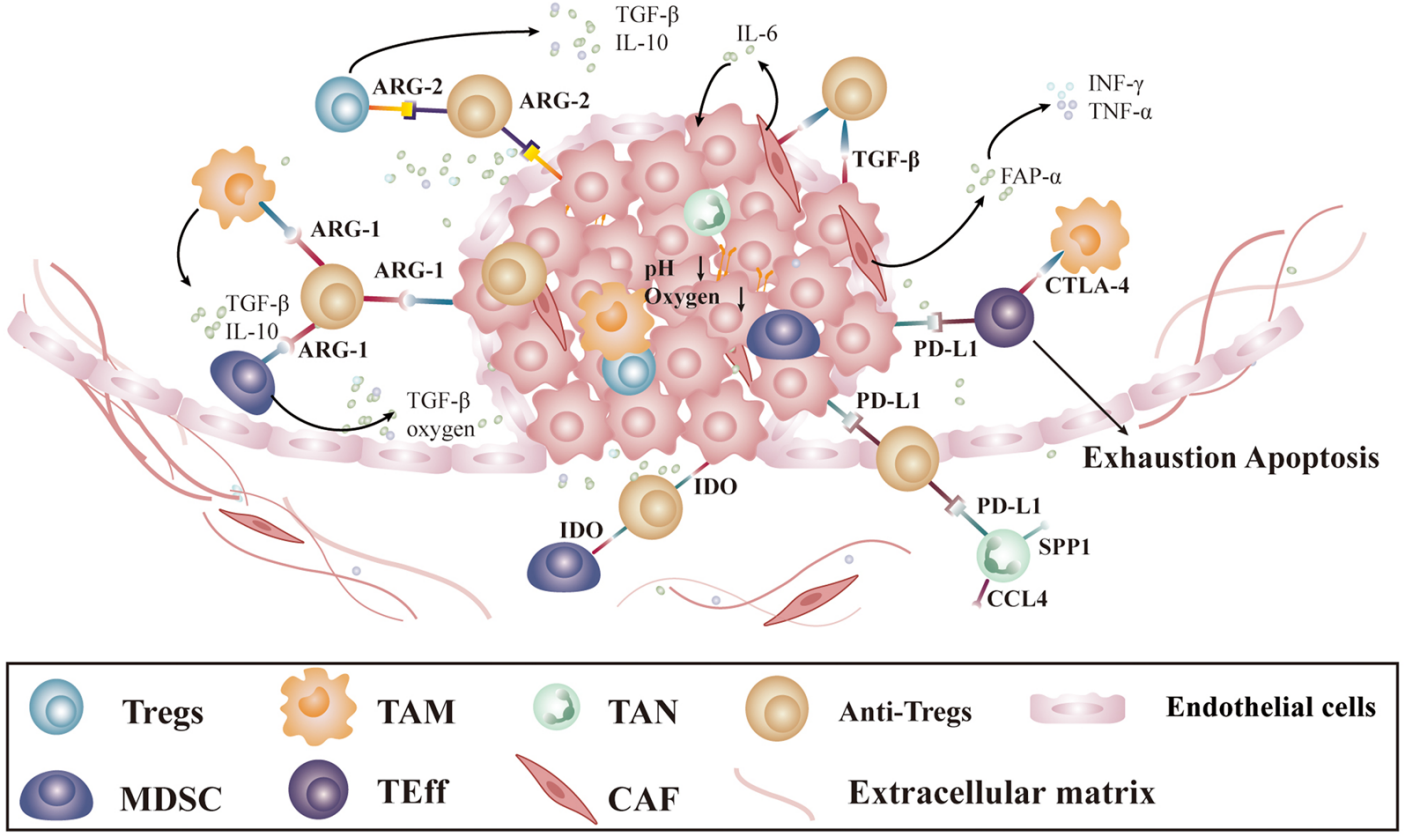
The immunosuppressive tumor microenvironment hinders CAR T-cell therapy for solid tumors. The solid tumor microenvironment is a complex and evolving entity containing immune-suppressing cells such as Tregs, MDSCs, TAMs and TANs; there are also CAFs, endothelial cells and extracellular matrix. The immunosuppressive ligands PD-L1, ARG-1, ARG-2, CTLA-4, and IDO secreted by those cells in the TME may all quell the intrinsic antitumor immune response, as well as the CAR T-cell response that helps tumor cells evade immune cell attack. Targeting those TMAs not only leads to a direct attack on the tumor cells but also modulates the tumor microenvironment, rendering it immunocompetent and tumor-hostile. *Tregs* regulatory T cells; *TAM* tumor-associated macrophages; *TAN* tumor-associated neutrophils; *Anti-Tregs* anti-regulatory T cells; *MDSC* myeloid-derived suppressor cells; *TEff* effector T cells; *CAF* cancer-associated fibroblasts. *PD-L1* programmed cell death 1 ligand 1; *IDO* indoleamine 2,3-dioxygenase; *ARG-1* arginase 1; *ARG2* arginase 2; *CTLA-4* cytotoxic T-lymphocyte-associated protein 4

​ Suppressive immune cells, such as regulatory T cells (Tregs) and myeloid-derived suppressor cells (MDSCs), in the tumor microenvironment can secrete large amounts of PEG2, TGF-β, IL-10, nitric acid, and indoleamine 2, 3-dioxygenase (IDO) to inhibit T-cell proliferation. Immunosuppressive ligands, such as programmed cell death 1 ligand 1 (PD-L1), might induce intrinsic antitumor immune responses as well as CAR T-cell responses (Fig. 3) [84].

Immune checkpoint inhibitors such as CTL4 and PD1 combined with CAR T cells have been used to target immune cells and weaken the role of immune cells in the immune microenvironment [85]. The T-cell immunoglobulin mucin 3 (Tim3) is an immune checkpoint receptor that plays a dominant role in T-cell depletion in the tumor microenvironment. The Hadjati team knocked down Tim3 in MSLN CAR T cells, and the knockout CAR T-cell lines had improved cytotoxic function, enhanced cytokine production and proliferation, and altered the immune microenvironment in response to the mitigated Tim3 signaling pathway [86].

### Inefficient infiltration of CAR T cells in solid tumors

CAR-T cells have more opportunity to interact with blood tumor cells, whereas it is difficult for CAR-T cells to penetrate tumor tissues through the blood system in solid tumors. Furthermore, the lack of chemokine expression involved in T-cell infiltration of tumor tissues and the presence of dense fibrotic stroma in solid tumors result in a reduced ability of CAR-T cells to migrate and invade tumor cells [87]. Treatment with PARP inhibitors promoted CAR T-cell infiltration by stimulating a chemokine milieu and modulated the tumor microenvironment by activating the cGAS-STING pathway [88]. We can also eliminate poor tumor infiltration by decoding the complexities of IFN/PRR signaling and then engineering CAR T cells to deploy RN7SL1 to recode these signals in the tumor microenvironment. RN7SL1 CAR T cells exhibit good tumor infiltration, myeloid cell and DC production activation, a tumor microenvironment with antigen provision, and endogenous T cells to reject solid tumors under the threat of CAR antigen loss [89]. Furthermore, they can be reinfused within the tumor to improve CAR T-cell infiltration and the transshipment problem in some types of solid tumors.

### Chronic antigen stimulation leads to CAR T-cell exhaustion

Limited persistence remains a significant hindrance to the advancement of effective CAR T-cell therapy. This cellular state is characterized by the overexpression of PD-L1 and other inhibitory factors. T-cell exhaustion, which can lead to treatment failure and cancer recurrence, is likely to play a major role in CAR T-cell therapy. Not only tonic signaling but also chronic and persistent antigen stimulation can lead to variable levels of T-cell exhaustion. Compared to hematological malignancies, solid tumor macroenvironments challenge CAR T cells with hypoxic, acidic, and nutrient-poor conditions that alter metabolism and accelerate exhaustion [90].

Perturbation of the INO80 and BAF chromatin remodeling complexes improves T-cell persistence in tumors. Depletion of Arid1a, a BAF complex member, resulted in the maintenance of an effector program and downregulation of exhaustion-related genes in tumor-infiltrating T cells [91]. Another study showed that RASA2 and SynNotch CAR T cells can overcome the challenges of specificity, heterogeneity, and persistence [69, 92].

## The target of TMAs for CAR T-cell therapies in solid tumors

TMAs were first described by Andersen MH [93]. They include IDO, PD-L1, tryptophan 2,3-dioxygenase (TDO), arginase 1 and 2, CCL22, TGF β, and FoxP3, which are expressed by heterogeneous cell types in the tumor microenvironment, such as MDSCs, tumor-associated macrophages (TAMs), tumor-associated neutrophils (TANs), tumor-associated dendritic cells (DCs), Treg cells and cancer-associated fibroblasts (CAFs), that help tumor cells escape immune cell attack (Fig. 3) [93].

As therapeutic targets, TMAs offer several advantages that differentiate them from more traditional tumor antigens. ​Combined targeted TMAs for antitumor immunotherapy can directly attack tumor cells, modulate the tumor microenvironment, establish a microenvironment that is not conducive to tumor growth, modulate immune cells in the microenvironment, restore their immune activity and tumor-killing effect, and inhibit tumor angiogenesis. Although the concept of TMAs has not yet been proposed in CAR T-cell therapy, the use of TMAs as binding domains for CARs has been applied in the study of CAR T-cell therapy. Tong Li constructed PD1-anti-MUC16 dual-CAR T cells that released many cytokines, such as IL-2, IFN-γ, and TNF-α, which demonstrated enhanced killing capacity of OVCAR-3 cells compared to single CAR T cells in vivo and survived significantly longer in vivo [94]. Wenzhen Li constructed a second-generation CAR targeting EpCAM plus hsBCL9_CT_-24, a peptide that inhibits the β-catenin/BCL9 interaction and exhibits potent antitumor effects. The results indicated that targeting EpCAM and Wnt/β-catenin can inhibit tumor growth and enhance tumor infiltration without affecting T-cell survival, promoting the efficacy of EpCAM CAR T-cell therapy [57]. Researchers have generated bispecific CAR T cells targeting EpCAM and ICAM-1 to increase resistance to antigen escape. ICAM-1 can induce the secretion of proinflammatory cytokines upon CAR interaction with the primary antigen EpCAM; thus, tumor cells are more vulnerable to dual CAR T cells [55].

A recent study showed that third-generation HER2 CAR T cells could efficiently eliminate glioblastoma cells in vitro and promote CAR T antitumor activity when combined with PD-1 blockade [94]. Shaw AR increased the breadth, potency, and duration of anti-PDAC HER2-specific CAR T-cell activity with oncolytic adeno-immunotherapy, which produced cytokines, achieved immune checkpoint blockade, and had a safety switch (CAdTrio). The combined use of CAdTrio and HER2 cured tumors in two PDAC xenograft models and produced a lasting tumor response in humanized mice, making it a potential treatment regimen for PDAC patients beyond surgical treatment [95]. Li et al. constructed HER2 CAR T cells secreting IL-2 and IFN, which bound to anti-PD1 antibodies, impaired the enhanced growth of HCC1954 tumors and overcame the resistance of trastuzumab [96]. He et al. constructed a hypoxia-inducible transcription amplification system (HiTA system) to control CAR expression in HiTA-CAR T cells. This HiTA system showed a significant improvement in hypoxia-restricted transgene expression over existing systems and showed significant antitumor activity in the absence of significant hepatic or systemic toxicity observed in vivo. This approach can also be applied to the design of CAR T cells directed against other tumor antigens [97]. Deepak et al. developed “switch” CAR systems, the activity of which could be controlled in vivo and which bound specific peptides; the specific peptides bound to Fab molecules, and the Fab bound to tumors. The “switch” acts as a bridge between the target and effector cells and controls antigen specificity, activation, and eventual tumor clearance. These switches have the advantage of a short half-life of the Fab fragment and limited immunogenicity of the peptide tag. This switchable CAR T is effective in preclinical models of leukemia and breast cancer cells and may allow the safe use of HER2 in clinical settings [98].

Local intrahepatic perfusion optimized the delivery of CAR T cells to liver metastases, but Burga et al. expected liver CD11b + Gr-1 + myeloid-derived suppressive cells (L-MDSCs) to inhibit CAR T-cell delivery to the liver. ​Experimental validation demonstrated the recovery of CAR T-cell efficacy when mice were treated with CEA CAR T cells in combination with L-MDSC depletion and GM-CSF neutralization by inhibition of L-MDSC expansion or PD-L1 blockade. In the future, inhibiting L-MDSC amplification after CEA CAR T-cell treatment will be a new research direction [99].

​At the same time, the challenges of insufficient T-cell penetration and weak effector function in the application of TMAs in CAR T-cell therapy should also be addressed. Therefore, TMAs should be combined with other immunotherapies to achieve better clinical efficacy, such as degradation of the tumor extracellular matrix using matrix metalloproteinase, which can help CAR T cells infiltrate tumors [100].

## Conclusion

Humans have been fighting cancer for thousands of years, but the incidence of cancer continues to increase, and we are constantly searching for new cancer treatments. CAR T cells have occupied a leading position since their inception, from their application against hematological malignancies to many attempts to treat solid tumors. Scientists have identified several TAAs of solid tumors, some of which have been tested in phase I and II clinical trials. CAR T-cell treatment has encountered many problems in the treatment of solid tumors, and scientists have also worked out various solutions. In future studies, on the one hand, it is vital to increase multitarget CAR T-cell development to enhance their tumor-killing specificity and reduce off-target effects so that CAR T cells can simultaneously recognize the presence of two or more TAAs on tumors [61, 68, 75, 81, 101–103]. Dual CAR and tandem CAR can also be designed to enhance signaling by the 4-1BB and CD28 domains, optimized by the membrane-embedded proximal positioning of both signaling domains within separate, parallel CARs [104]. On the other hand, we developed a new strategy that combined targeting TMAs to create a tumor-hostile microenvironment and improve CAR T-cell infiltration into solid tumors. Third, it is necessary to construct a “suicide gene” system to destroy infused CAR T cells to regulate their proliferation and restrict their toxicity [72, 105, 106] or to construct a “device switch” to regulate the timing of T-cell activation to prevent emerging CRS and CERS and to moderate the apoptosis of CAR T cells [95, 98, 107–110].

Moreover, universal CAR T cells could be constructed for specific tumors by targeting highly expressed specific targets, which will shorten the waiting time, reduce the cost, broaden the cell source, strengthen the operability and quality control for patients, and make it easier to achieve widespread use. Nevertheless, universal CAR T cells should be given special attention to avoid GVHD compared with the side effects of autologous CAR T cells [111, 112]. The functions of solid tumor CAR T cells are different from those of hematological malignancy CAR T cells, implying that enhancing the binding interactions between CAR T cells and tumor cells may increase their responses in solid tumors [113]. New research has indicated that artificial thymic organoids allow the selective differentiation of CRT-transduced human iPSCs into CAR T cells and can have effective results in an animal model. This breakthrough may change the way T cells are obtained; it requires the patient’s blood to select CAR T cells [114, 115]. Researchers all over the world are trying to enhance the effectiveness and safety of CAR T-cell therapy, and we believe that it will be a big step toward curing cancer.

## Data Availability

Not applicable.

## Abbreviations

CAR T: Chimeric antigen receptor T
TAAs: Tumor-associated antigens
TMAs: Tumor microenvironment antigens
TSA: Tumor-specific antigens
TCR: T-cell receptor
scFv: Single chain variable fragment
ICOS: Inducible T-cell costimulatory factor
DAP10: DNA X-activating protein
MUC1: Mucin-1
CDE: Center for Drug Evaluation
HER2: Human epidermal growth factor receptor 2
EGFR: Epidermal growth factor receptor
BiTE: Bispecific T-cell engager
CEA: Carcinoembryonic antigen
PET: Positron emission tomography
CT: Computed tomography
MSLN: Mesothelin
OVCA: Ovarian adenocarcinoma
NKG2D: Natural killer group 2D
EpCAM: Epithelial cell adhesion molecule
Tim3: T-cell immunoglobulin mucin 3
GPC3: Glypican 3
CRS: Cytokine release syndrome
GM-CSF: Granulocytic macrophage colony-stimulating factor
CRES: CAR T-related encephalopathy syndrome
UCAR T: Universal CAR T
HVGR: Host versus graft reaction
GVHD: graft-versus-host disease
ZFNs: Zinc-finger nucleases
TALENs: Transcription activator-like nucleases.
